# Supplementary figures and images for: VDR and PDIA3 Are Essential for Activation of Calcium Signaling and Membrane Response to 1,25(OH)2D3 in Squamous Cell Carcinoma Cells
Source: Cells. 2023 Dec 20;13(1):11. doi: 10.3390/cells13010011 (PMC10778127; doi:10.3390/cells13010011)

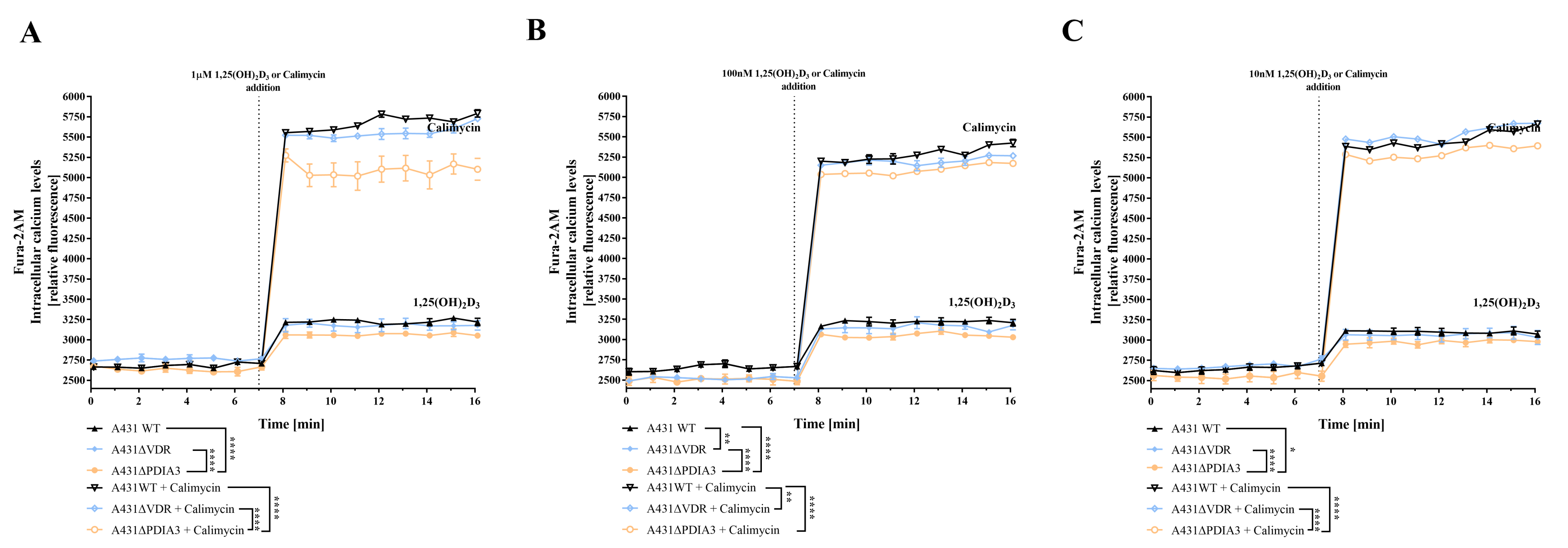

Supplement: Supplementary file 1 [file cells-13-00011-s001.zip › Supplementary figure S1.tiff]

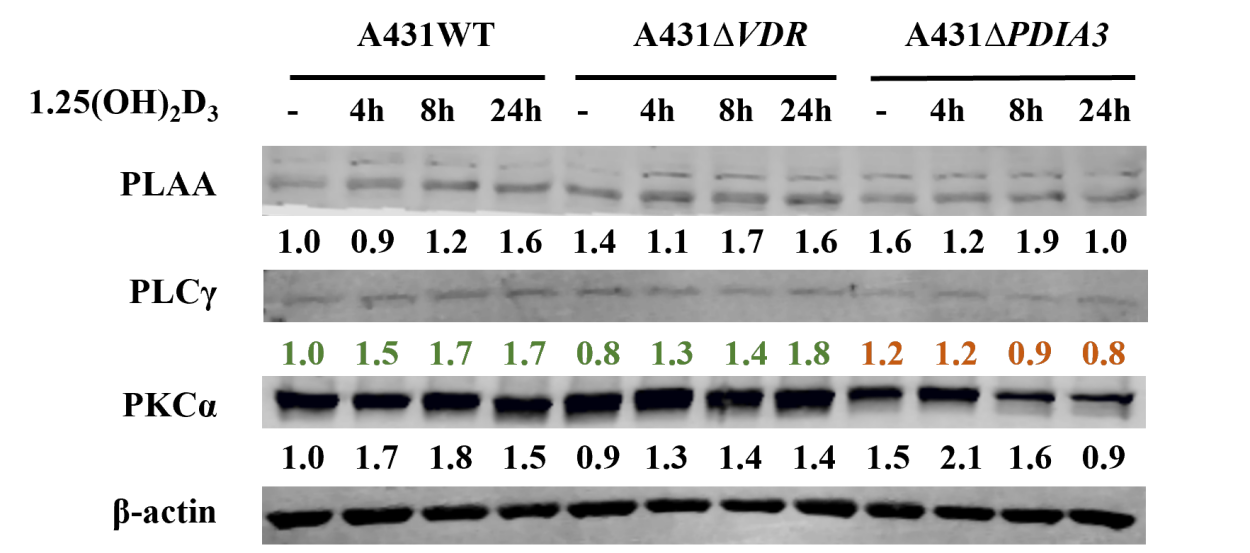

Supplement: Supplementary file 1 [file cells-13-00011-s001.zip › Supplementary figure S2.tiff]
